# Neurophysiological Differences between Flail Arm Syndrome and Amyotrophic Lateral Sclerosis

**DOI:** 10.1371/journal.pone.0127601

**Published:** 2015-06-09

**Authors:** Hecheng Yang, Mingsheng Liu, Xiaoguang Li, Bo Cui, Jia Fang, Liying Cui

**Affiliations:** Department of Neurology, Peking Union Medical College Hospital, Chinese Academy of Medical Sciences and Peking Union Medical College, Beijing, China; Institute of Health Science, CHINA

## Abstract

There are many clinical features of flail arm syndrome (FAS) that are different from amyotrophic lateral sclerosis (ALS), suggesting they are probably different entities. Studies on electrophysiological differences between them are limited at present, and still inconclusive. Therefore, we aimed to find clinical and neurophysiological differences between FAS and ALS. Eighteen healthy control subjects, six FAS patients and forty-one ALS patients were recruited. The upper motor neuron signs (UMNS), split-hand index (SI), resting motor threshold (RMT), central motor conduction time (CMCT) were evaluated and compared. There was no obvious upper motor neuron signs in FAS. The SI and RMT level in FAS was similar to control subjects, but significantly lower than that of in ALS. Compared with control group, the RMT and SI in ALS group were both significantly increased to higher level. However, no significant difference of CMCT was found between any two of these three groups. The differences in clinical and neurophysiological findings between FAS and ALS, argue against they are the same disease entity. Since there was no obvious UMNS, no split-hand phenomenon, and no obvious changes of RMT and CMCT in FAS patients, the development of FAS might be probably not originated from motor cortex.

## Introduction

Flail arm syndrome (FAS), also called man-in-barrel syndrome, or brachial amyotrophic diplegia, is a slowly progressive sporadic motor neuron disorder, characterized by severe flaccid paralysis and muscle wasting in both arms symmetrically, while relatively sparing the legs and bulbar parts, and with few signs of upper motor neuron lesions[1]. Amyotrophic lateral sclerosis (ALS) is another degenerative motor neuron disorder, with progressive loss of both upper and lower motor neurons in motor cortex, spinal anterior horn cells and motor neurons in brain stem[2].

The relationship between FAS and ALS is still unclear. Some researchers argue this syndrome is a variant of ALS, while others believe that FAS is an independent entity[3–5]. There are several clinical characteristics of FAS that are very different from classical ALS, including male predominance, the involvement of proximal muscles of both arms, longer natural history[6]. Previously, this syndrome was categorized in the progressive muscular atrophy (PMA) group because of relatively few impairment of upper motor neurons. However, the natural history and sexual predominance of FAS are different from that of PMA as well[6]. These obviously different clinical features suggest that FAS might be a different entity different from either classical ALS, or PMA.

Concerning the differences of neurophysiological investigations between FAS and ALS, there were limited studies and the evidence was still inconclusive. In this study, the clinical features, split-hand index (SI), excitability of motor cortex (resting motor threshold, RMT), and central motor conduction time (CMCT) were documented in both FAS and classical ALS patients in order to detect the electrophysiological differences between them.

## Materials and Methods

### Subjects

Six FAS patients, forty-one sporadic ALS patients and eighteen healthy volunteers were recruited for this study from March 2013 to March 2015. The diagnosis of FAS was made when patients presented progressive muscle weakness and wasting in both upper extremities, especially the arms, without functionally involving other parts such as legs, or bulbar muscles for at least 18 months from the disease onset. All ALS patients met revised El Escorial research diagnostic criteria for clinically definite or probable ALS[7]. The inclusion criteria: those FAS or ALS patients who agreed to participate in this study; healthy volunteers with age of 30–65 years old. The exclusion criteria: those participants with complications that contraindicated of TMS study, including seizure attacks or implantation of cardiac pacemakers. This study was approved by the ethical review board of Peking Union Medical College Hospital and the written informed contents from all the participants were obtained.

The upper motor neuron lesion was evaluated clinically in all ALS and FAS patients. The clinical UMNS were defined as: definite (extensor plantar responses, hyperreflexia, or spasticity), probable (clonus, brisk reflexes in a weak extremity, hyperactive jaw jerk, or Hoffman’s signs, without extensor plantar responses or spasticity), modified according to previous criteria[8].

### Split-hand index

The routine motor nerve conduction studies of bilateral median and ulnar nerves were performed in all participants. The band pass filter of EMG machine (Key point, Denmark) was set between 2Hz and 10kHz. The compound muscle action potentials (CMAPs) of abductor pollicis brevis (APBs) and abductor digiti minimi (ADMs) were recorded using pairs of surface electrodes, with recording electrode placing on the belly of muscles while reference electrode on the distal tendons. The peak-to-peak amplitudes of CMAPs were documented with supramaximal electronic stimulus of median and ulnar nerves. The split-hand index (SI) was calculated by dividing the CMAP amplitude of ADMs by that of APBs. The maximum SI of two sides was chose to be represented as the SI of one individual.

### Resting motor threshold

The motor evoked potentials (MEPs) were performed using a figure “8”-shaped magnetic coil with diameter of 96mm, connecting with transcranial magnetic stimulator (Magventure, Magpro, Denmark) with maximal stimulus output (MSO) intensity of 2.2 Tesla. The band pass filter was set in the same way as in motor nerve conduction studies. The pairs of surface electrodes for documenting MEPs of ADMs were the same pairs used in motor nerve conduction studies. The RMT level was equal to the minimal magnetic stimulus intensity required to evoke the responses with peak-to-peak amplitude of at least 50 μV in at least three times in six consecutive trials[9]. The RMT tested on the upper extremity that was involved earliest in ALS and FAS patients was investigated.

### Central motor conduction time

CMCT was calculated by subtracting the peripheral nerve part from the total latency of response elicited on motor cortex[9]. The foraminal electromagnetic stimulation method was used to measure the peripheral motor conduction time. The center of magnetic coil was placed over C7 cervical spine to stimulate cervical nerve roots of lower segment to acquire the peripheral part of latency of MEPs responses. The MEPs responses were elicited by placing coil over the optimal stimulating sites of motor cortexes, and the MEPs total latency was documented. CMCT = MEPs total latency—peripheral motor conduction time.

### Statistical analysis

These data were expressed as means ± standard errors of means. The Kruskal-Wallis tests were used to compare the difference of SI, RMT and CMCT among three groups, and the Nemeyi tests were used to compare the statistical differences between any of two groups. A *p* value <0.05 was denoted to be statistically significant.

## Results

### Clinical profiles of subjects in our study

Table 1 showed the clinical and demographic features of our subjects in this study. There was obvious male predominance in the FAS patients, with only one female patient in six FAS patients, very different from that of in ALS patients. In ALS group, the ratio of female to male was 1:1.56 (16:25). Moreover, there was another difference between FAS and ALS patients in the presence of UMNS. None of FAS patients showed definite or probable UMNS. In contrast, 31 out of 41ALS patients presented definite UMNS with or without probable UMNS, while the remainders of 10 ALS patients showed probable UMNS.

**Table 1:** The clinical and demographic characteristics of subjects in this study.

	**control**	**FAS**	**ALS**
****Total (F:M)****	18 (8:10)	6 (1:5)	41 (16:25)
****Age (year)****	44.8±2.1	51.0±4.7	50.0±1.4
****Definite UMNS****	0	0	31
****Probable UMNS****	0	0	10

### Changes of split-hand index in FAS and ALS patients

**Fig 1:**
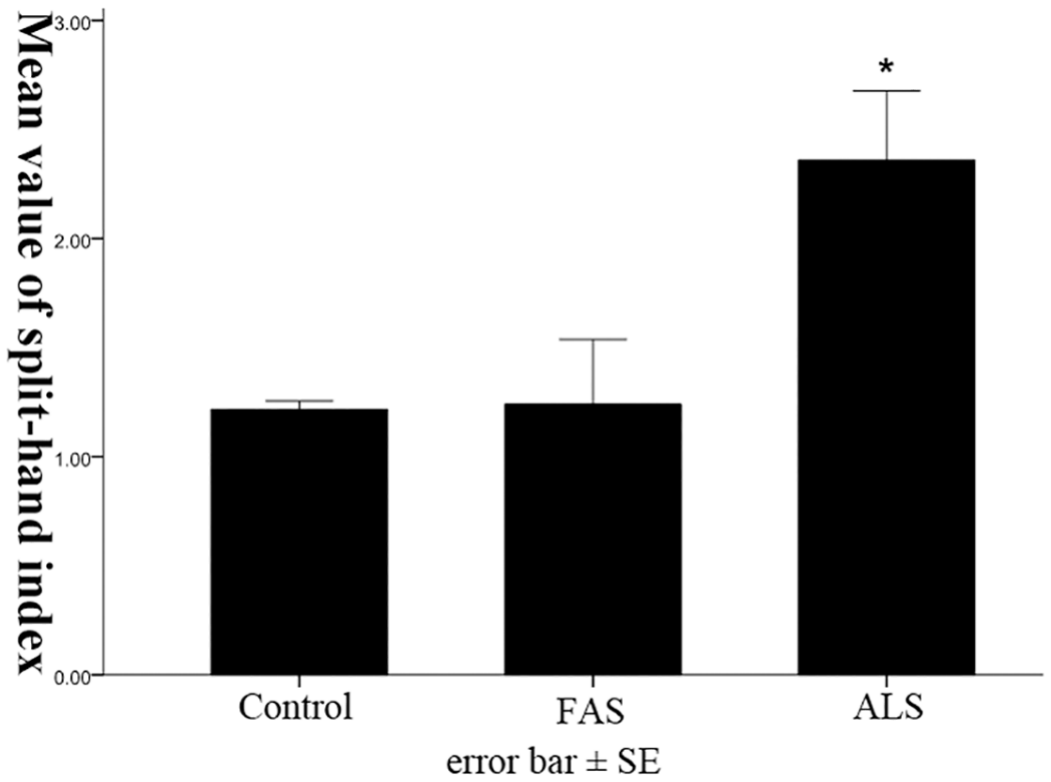
Changes of split-hand index in FAS and ALS patients. The SI in FAS patients was increased slightly without significance, compared with control, but was significantly lower than that of in ALS patients (*p<0.05).

The mean value of SI in control people was 1.22±0.04, while those in FAS group and ALS groups were 1.24±0.30, 2.36±0.32, respectively. There was no significant difference of SI between FAS group and health control (Fig 1, *p* = 0.849). The SI of ALS patients was significantly higher, compared with that of FAS or control subjects (*p*<0.05).

### Differences of RMT between three groups

Compared with control group, there was no statistical difference of RMT in FAS patients (*p* = 0.841), but the level of RMT was significantly higher in ALS (*p*<0.05). Moreover, the RMT in ALS patients was also significantly higher than that of in FAS patients (*p*<0.05).

**Fig 2:**
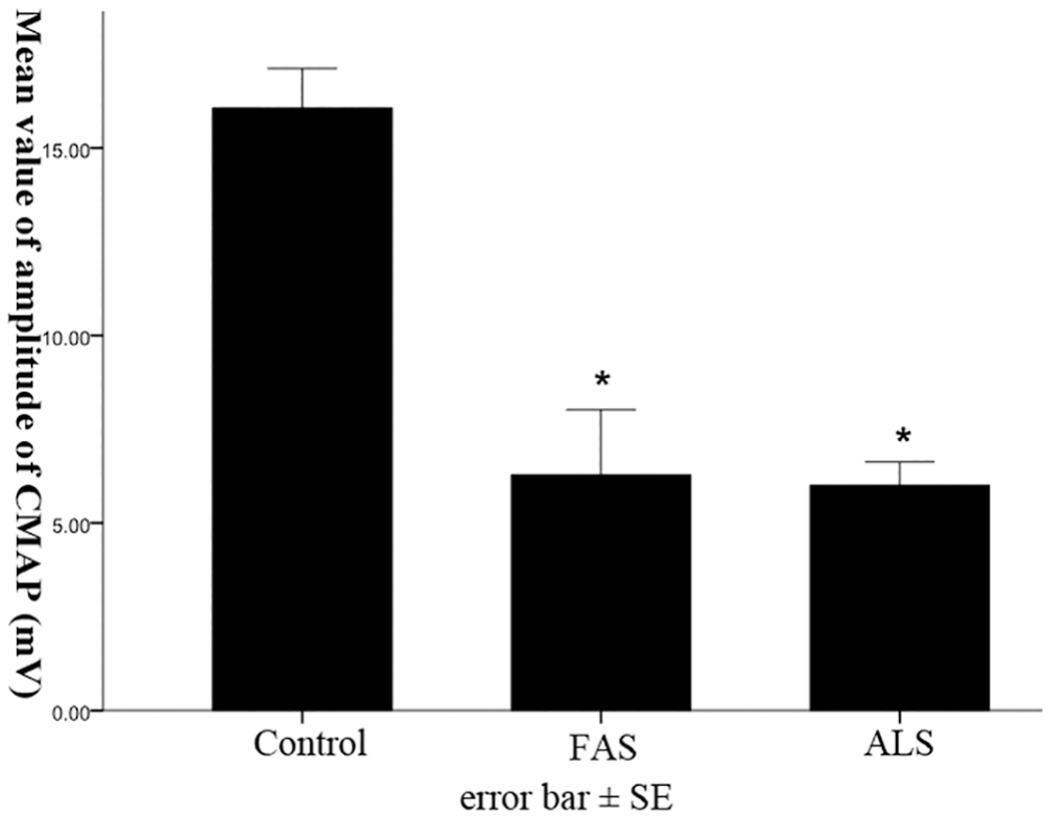
Difference of the amplitude of CMAPs of ADMs between three groups. Fig 2 showed the CMAPs amplitude of ADMs in ALS and FAS groups were significantly decreased, compared with control group (*p<0.05).

The CMAPs amplitudes of ADMs in FAS (6.3±1.7 mV) and ALS (6.0±0.6 mV) group were significantly lower, compared with control people (16.1±1.1 mV) (*p*<0.05). Although the CMAPs amplitudes in FAS were lower than that of ALS, there was no statistical significance between them (Fig 2, *p* = 0.706).

### Comparisons of CMCT among three groups

**Fig 3:**
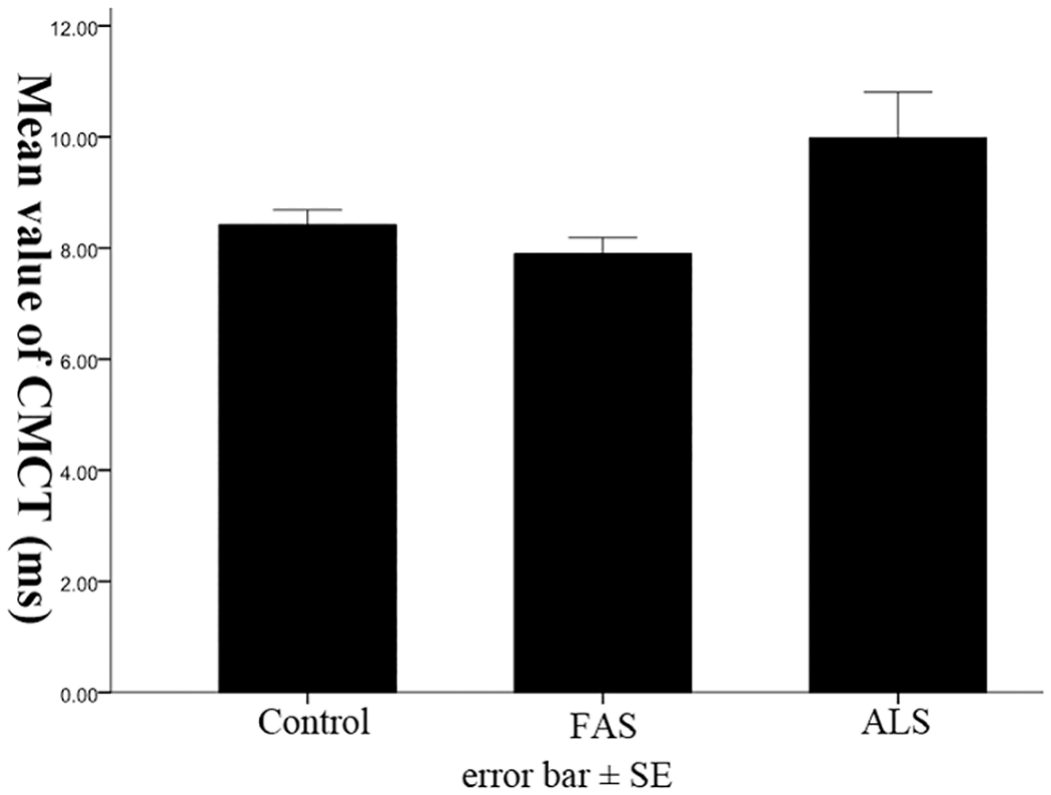
Comparison of CMCT among three groups. The CMCT of FAS and ALS patients was not significantly changed, compared with control.

The mean value of CMCT in healthy control was 8.4±0.3ms, while that of in FAS and ALS was 7.9±0.29ms, and 10.0±0.8ms, respectively. There was no significant difference of CMCT among these three groups (Fig 3, *p* = 0.138).

## Discussion

In this study, we found that there was no significant difference of SI, RMT, CMCT between FAS patients and control subjects. However, there were significant differences between FAS and ALS patients, in terms of clinical features, as well as of neurophysiological studies. The significantly higher SI, and increased RMT in ALS were distinctive neurophysiological features that were distinguishable from FAS.

There are several clinical aspects that can distinguish FAS and ALS patients from each other. FAS has male predominance, with a ratio of male to female being 4~10:1, which is only 1.1~1.5:1 in ALS patients[6, 10]. In our study, we have the very similar findings, confirming that male predominance is an important feature of FAS, though the underlying mechanism is still to be determined. FAS is characterized by relatively symmetric involvement of proximal muscles of both arms without obvious muscle weakness of legs or bulbar sites[6, 10]. The prognosis of FAS is significantly better than that of ALS, in terms of median survival rates, as well as five-year survival rates[6]. These six FAS patients in this study progressed very slowly, and the symptoms and signs were still confined to bilateral upper extremities 18 months after disease onset.

Another obvious difference between FAS and ALS is the involvement of upper motor neurons. The upper motor neuron lesion might be slight in FAS patients, if any, because there was usually no obvious UMNS in most FAS patients, such as hypereflexia, hypertonia, or pathological signs[1, 11]. In this study, none of six FAS patients has definite or probable UMNS clinically. Although some of them show higher RMT level, the obvious involvement of lower motor neurons may be the leading cause. It was reported that about 70% FAS patients developed UMNS in the lower extremities[12], and 50% FAS patients showed UMNS in the upper extremities[13]. The occurrence rate of UMNS in FAS patients seems strikingly high, very different from other studies and our result[1, 3, 11]. One of reasons might be that different operational definitions for FAS were adopted. Therefore, some upper limb onset ALS patients might be misdiagnosed as FAS, since there was no definite criteria of how long the functional involvement must be confined to the flail arms to make the diagnosis of FAS. The other possible reason might be that the definitions of UMNS were different in these studies. Although it was still unclear whether these six FAS patients would develop UMNS in the future, they did not show any definite or probable UMNS at the time of analysis. In previous study, post mortem findings of one patient diagnosed with “FAS” revealed evidences of upper motor neuron lesions showing degeneration of the pyramidal tracts and motor cortex, accompanied with loss of spinal anterior horn cells and motor neurons of brain stem; while these changes were not found in another FAS patient, except obvious loss of spinal anterior horn cells[11]. The initial diagnosis of FAS in the first patient seems doubtful, because of relatively early involvement of bulbar muscles (13 months after disease onset). Therefore, it might be too early to believe that there are sufficient definite pathological evidences of obvious involvement of pyramidal tracts, motor cortex in FAS patients.

Although the causes and definite mechanisms of ALS are still unclear, ALS is considered to be a disorder of motor cortex, and the excitotoxic effects on motor neurons might underlie the development of this disease[14, 15]. Previous studies show the level of excitatory neurotransmitter in cortex and CSF is increased, and hyper-excitability of motor cortex is an important feature in early ALS[16–20]. The hyper-excitability of cortical motor neurons supposedly produce metabolic impairment of lower motor neurons projected, and lead to both upper and lower motor neurons injury[14]. Split-hand phenomenon is one important clinical feature of ALS patients, characterized by preferential wasting of the thenar muscles, while the hypothenar muscles are relatively less involved[21–23]. Our findings showing significantly increased SI in ALS, are consistent with previous studies, and suggest that split-hand is an important neurophysiological feature of ALS[22, 24–26]. Since these muscles of thenar and hypothenar were innervated by the same myotomes, the difference of involvement severity of these two sites seems not be derived from spinal cord. The cortical motor neuronal input to the thenar spinal pool is more severely impaired than that of the hypothenar spinal pool in ALS, suggesting the preferential involvement of thenar muscles having a cortical basis[21, 23]. These results provide important evidences supporting that the development of ALS might be originated from motor cortex.

In contrast, there seems little involvement of motor cortex in FAS patients. MRI investigations reveal no obvious thinning of primary motor cortex in pure lower motor neuron disorders, including FAS, while significant thinning of primary motor cortex is found in classical ALS[27]. Moreover, the split-hand phenomenon seems not occur in FAS patients. There is no significant increase of SI in FAS patients, very different from ALS in our study. Our result is consistent with previous research, showing that the split-hand phenomenon is not commonly seen in FAS[25]. Therefore, the weakness and atrophy of hands in FAS might not have a cortical basis. These results indicate that FAS might have a very different pathophysiological basis from ALS. Previous research show the RMT level and short intracortical interval (SICI) in eight out of eleven FAS patients is significantly reduced, suggesting of increased excitability of motor cortex in FAS, which is similar to electrophysiological changes in ALS[3]. However, we did not find obvious increase or decrease of RMT in our FAS patients in this study.

In summary, there is limited evidence showing that upper motor neurons are obviously damaged in FAS patients. Moreover, FAS does not share many clinical and electrophysiological features in common with ALS, arguing for that they might be two different disease entities. As for the drug trials and drug treatment in the future, our study indicates that FAS patients should be separated from ALS patients. However, the results should be interpreted cautiously, because of relatively small sample size of FAS patients in this study.

## Supporting Information

S1 File The STROBE checklist of this study. (DOCX)
