# Supplementary material for: Neurophysiological Differences between Flail Arm Syndrome and Amyotrophic Lateral Sclerosis
Source: PLoS One. 2015 Jun 9;10(6):e0127601. doi: 10.1371/journal.pone.0127601 (PMC4461255; doi:10.1371/journal.pone.0127601)
Supplement: S1 File — (DOCX) [file pone.0127601.s001.docx]

S1. The STROBE checklist of this study.

|  | Item No. | Recommendation | Page  No. | Relevant text from manuscript |
| --- | --- | --- | --- | --- |
| **Title and abstract** | 1 | (*a*) Indicate the study’s design with a commonly used term in the title or the abstract | 1 |  |
|  |  | (*b*) Provide in the abstract an informative and balanced summary of what was done and what was found | 1 | Eighteen healthy control subjects, six FAS patients and forty-one ALS patients were recruited. The upper motor neuron signs (UMNS), split-hand index (SI), resting motor threshold (RMT), central motor conduction time (CMCT) were evaluated and compared. There was no obvious upper motor neuron signs in FAS. The SI and RMT level in FAS was similar to control subjects, but significantly lower than that of in ALS. Compared with control group, the RMT and SI in ALS group were both significantly increased to higher level. However, no significant difference of CMCT was found between any two of these three groups. |
| Introduction | | | |  |
| Background/rationale | 2 | Explain the scientific background and rationale for the investigation being reported | 2 | Flail arm syndrome (FAS), also called man-in-barrel syndrome, or brachial amyotrophic diplegia, is a slowly progressive sporadic motor neuron disorder, characterized by severe flaccid paralysis and muscle wasting in both arms symmetrically, while relatively sparing the legs and bulbar parts, and with few signs of upper motor neuron lesions[1]. Amyotrophic lateral sclerosis (ALS) is another degenerative motor neuron disorder, with progressive loss of both upper and lower motor neurons in motor cortex, spinal anterior horn cells and motor neurons in brain stem[2].  The relationship between FAS and ALS is still unclear. Some researchers argue this syndrome is a variant of ALS, while others believe that FAS is an independent entity[3-5]. There are several clinical characteristics of FAS that are very different from classical ALS, including male predominance, the involvement of proximal muscles of both arms, longer natural history[6]. Previously, this syndrome was categorized in the progressive muscular atrophy (PMA) group because of relatively few impairment of upper motor neurons. However, the natural history and sexual predominance of FAS are different from that of PMA as well[6]. These obviously different clinical features suggest that FAS might be a different entity different from either classical ALS, or PMA. |
| Objectives | 3 | State specific objectives, including any prespecified hypotheses | 2 | Concerning the differences of neurophysiological investigations between FAS and ALS, there were limited studies and the evidence was still inconclusive. In this study, the clinical features, split-hand index (SI), excitability of motor cortex (resting motor threshold, RMT), and central motor conduction time (CMCT) were documented in both FAS and classical ALS patients in order to detect the electrophysiological differences between them. |
| Methods | | | |  |
| Study design | 4 | Present key elements of study design early in the paper | N/A |  |
| Setting | 5 | Describe the setting, locations, and relevant dates, including periods of recruitment, exposure, follow-up, and data collection | 2-3 | Six FAS patients, forty-one sporadic ALS patients and eighteen healthy volunteers were recruited for this study from March 2013 to March 2015. The inclusion criteria: those FAS or ALS patients who agreed to participate in this study; healthy volunteers with age of 30-65 years old. The exclusion criteria: those participants with complications that contraindicate of TMS study, including seizure attacks or implantation of cardiac pacemakers. This study was approved by the ethical review board of Peking Union Medical College Hospital and the written informed contents from all the participants were obtained. |
| Participants | 6 | (*a*) *Cohort study*—Give the eligibility criteria, and the sources and methods of selection of participants. Describe methods of follow-up  *Case-control study*—Give the eligibility criteria, and the sources and methods of case ascertainment and control selection. Give the rationale for the choice of cases and controls  *Cross-sectional study*—Give the eligibility criteria, and the sources and methods of selection of participants | N/A |  |
|  |  | (*b*)*Cohort study*—For matched studies, give matching criteria and number of exposed and unexposed  *Case-control study*—For matched studies, give matching criteria and the number of controls per case | N/A |  |
| Variables | 7 | Clearly define all outcomes, exposures, predictors, potential confounders, and effect modifiers. Give diagnostic criteria, if applicable | 2-3 | The diagnosis of FAS was made when patients presented progressive muscle weakness and wasting in both upper extremities, especially the arms, without functionally involving other parts such as legs, or bulbar muscles for at least 18 months from the disease onset. All ALS patients met revised El Escorial research diagnostic criteria for clinically definite or probable ALS[7]. |
| Data sources/measurement | 8* | For each variable of interest, give sources of data and details of methods of assessment (measurement). Describe comparability of assessment methods if there is more than one group | *3-4* | **Split-hand index**  The routine motor nerve conduction studies of bilateral median and ulnar nerves were performed in all participants. The band pass filter of EMG machine (Key point, Denmark) was set between 2Hz and 10kHz. The compound muscle action potentials (CMAPs) of abductor pollicis brevis (APBs) and abductor digiti minimi (ADMs) were recorded using pairs of surface electrodes, with recording electrode placing on the belly of muscles while reference electrode on the distal tendons. The peak-to-peak amplitudes of CMAPs were documented with supramaximal electronic stimulus of median and ulnar nerves. The split-hand index (SI) was calculated by dividing the CMAP amplitude of ADMs by that of APBs. **Resting motor threshold**  The motor evoked potentials (MEPs) were performed using a figure “8”-shaped magnetic coil with diameter of 96mm, connecting with transcranial magnetic stimulator (Magventure, Magpro, Denmark) with maximal stimulus output (MSO) intensity of 2.2 Tesla. The band pass filter was set in the same way as in motor nerve conduction studies. The pairs of surface electrodes for documenting MEPs of ADMs were the same pairs used in motor nerve conduction studies. The RMT level was equal to the minimal magnetic stimulus intensity required to evoke the responses with peak-to-peak amplitude of at least 50 μV in at least three times in six consecutive trials[8]. The RMT tested on the upper extremity that was involved earliest in ALS and FAS patients was investigated.  **Central motor conduction time**  CMCT was calculated by subtracting the peripheral nerve part from the total latency of response elicited on motor cortex[8]. The foraminal electromagnetic stimulation method was used to measure the peripheral motor conduction time. The center of magnetic coil was placed over C7 cervical spine to stimulate cervical nerve roots of lower segment to acquire the peripheral part of latency of MEPs responses. The MEPs responses were elicited by placing coil over the optimal stimulating sites of motor cortexes, and the MEPs total latency was documented. CMCT= MEPs total latency – peripheral motor conduction time. |
| Bias | 9 | Describe any efforts to address potential sources of bias | N/A |  |
| Study size | 10 | Explain how the study size was arrived at | N/A |  |

Continued on next page

| Quantitative variables | 11 | Explain how quantitative variables were handled in the analyses. If applicable, describe which groupings were chosen and why | 3-4 | The maximum SI of two sides was chose to be represented as the SI of one individual.  The RMT tested on the upper extremity that was involved earliest in ALS and FAS patients was investigated. |
| --- | --- | --- | --- | --- |
| Statistical methods | 12 | (*a*) Describe all statistical methods, including those used to control for confounding | 4 | These data were expressed as means ± standard errors of means. The Kruskal-Wallis tests were used to compare the difference of SI, RMT and CMCT among three groups, and A *p* value <0.05 was denoted to be statistically significant. |
|  |  | (*b*) Describe any methods used to examine subgroups and interactions | 4 | the Nemeyi tests were used to compare the statistical differences between any of two groups. |
|  |  | (*c*) Explain how missing data were addressed | N/A |  |
|  |  | (*d*) *Cohort study*—If applicable, explain how loss to follow-up was addressed  *Case-control study*—If applicable, explain how matching of cases and controls was addressed  *Cross-sectional study*—If applicable, describe analytical methods taking account of sampling strategy | N/A |  |
|  |  | (*e*) Describe any sensitivity analyses | N/A |  |
| Results | | | | |
| Participants | 13* | (a) Report numbers of individuals at each stage of study—eg numbers potentially eligible, examined for eligibility, confirmed eligible, included in the study, completing follow-up, and analysed | N/A |  |
|  |  | (b) Give reasons for non-participation at each stage | N/A |  |
|  |  | (c) Consider use of a flow diagram | N/A |  |
| Descriptive data | 14* | (a) Give characteristics of study participants (eg demographic, clinical, social) and information on exposures and potential confounders | 4 | Table 1 showed the clinical and demographic features of our subjects in this study. There was obvious male predominance in the FAS patients, with only one female patient in six FAS patients, very different from that of in ALS patients. In ALS group, the ratio of female to male was 1:1.56 (16:25). Moreover, there was another difference between FAS and ALS patients in the presence of UMNS. None of FAS patients showed definite or probable UMNS. In contrast, 31 out of 41 ALS patients presented definite UMNS with or without probable UMNS, while the remainders of 10 ALS patients showed probable UMNS. |
|  |  | (b) Indicate number of participants with missing data for each variable of interest | N/A |  |
|  |  | (c) *Cohort study*—Summarise follow-up time (eg, average and total amount) | N/A |  |
| Outcome data | 15* | *Cohort study*—Report numbers of outcome events or summary measures over time | N/A |  |
|  |  | *Case-control study—*Report numbers in each exposure category, or summary measures of exposure | N/A |  |
|  |  | *Cross-sectional study—*Report numbers of outcome events or summary measures | N/A |  |
| Main results | 16 | (*a*) Give unadjusted estimates and, if applicable, confounder-adjusted estimates and their precision (eg, 95% confidence interval). Make clear which confounders were adjusted for and why they were included | 4-5 | **Changes of split-hand index in FAS and ALS patients**  The mean value of SI in control people was 1.22±0.04, while those in FAS group and ALS groups were 1.24±0.30, 2.36±0.32, respectively. There was no significant difference of SI between FAS group and health control (figure 1, *p*=0.849). The SI of ALS patients was significantly higher, compared with that of FAS or control subjects (*p*<0.05).  **Differences of RMT between three groups**  Compared with control group, there was no statistical difference of RMT in FAS patients (*p*=0.841), but the level of RMT was significantly higher in ALS (*p*<0.05). Moreover, the RMT in ALS patients was also significantly higher than that of in FAS patients (*p*<0.05).  The CMAPs amplitudes of ADMs in FAS (6.3±1.7 mV) and ALS (6.0±0.6 mV) group were significantly lower, compared with control people (16.1±1.1 mV) (*p*<0.05). Although the CMAPs amplitudes in FAS were lower than that of ALS, there was no statistical significance between them (figure 2, *p*=0.706).  **Comparisons of CMCT among three groups**  The mean value of CMCT in healthy control was 8.4±0.3ms, while that of in FAS and ALS was 7.9±0.29ms, and 10.0±0.8ms, respectively. There was no significant difference of CMCT among these three groups (*p*=0.138). |
|  |  | (*b*) Report category boundaries when continuous variables were categorized | N/A |  |
|  |  | (*c*) If relevant, consider translating estimates of relative risk into absolute risk for a meaningful time period | N/A |  |

Continued on next page

| Other analyses | 17 | Report other analyses done—eg analyses of subgroups and interactions, and sensitivity analyses | 4-5 |  |
| --- | --- | --- | --- | --- |
| Discussion | | | | |
| Key results | 18 | Summarise key results with reference to study objectives | 5 | In this study, we found that there was no significant difference of SI, RMT, CMCT between FAS patients and control subjects. However, there were significant differences between FAS and ALS patients, in terms of clinical features, as well as of neurophysiological studies. The significantly higher SI, and increased RMT in ALS were distinctive neurophysiological features that were distinguishable from FAS. |
| Limitations | 19 | Discuss limitations of the study, taking into account sources of potential bias or imprecision. Discuss both direction and magnitude of any potential bias | 8 | However, the results should be interpreted cautiously, because of relatively small sample size of FAS patients in this study. |
| Interpretation | 20 | Give a cautious overall interpretation of results considering objectives, limitations, multiplicity of analyses, results from similar studies, and other relevant evidence | 5-8 |  |
| Generalisability | 21 | Discuss the generalisability (external validity) of the study results | 8 | here is limited evidence showing that upper motor neurons are obviously damaged in FAS patients. Moreover, FAS does not share many clinical and electrophysiological features in common with ALS, arguing for that they might be two different disease entities. As for the drug trials and drug treatment in the future, our study indicates that FAS patients should be separated from ALS patients. |
| Other information | |  | | |
| Funding | 22 | Give the source of funding and the role of the funders for the present study and, if applicable, for the original study on which the present article is based | Cover letter |  |

*Give information separately for cases and controls in case-control studies and, if applicable, for exposed and unexposed groups in cohort and cross-sectional studies.

**Note:** An Explanation and Elaboration article discusses each checklist item and gives methodological background and published examples of transparent reporting. The STROBE checklist is best used in conjunction with this article (freely available on the Web sites of PLoS Medicine at http://www.plosmedicine.org/, Annals of Internal Medicine at http://www.annals.org/, and Epidemiology at http://www.epidem.com/). Information on the STROBE Initiative is available at www.strobe-statement.org.
